# Role of Probiotics in Halitosis of Oral Origin: A Systematic Review and Meta-Analysis of Randomized Clinical Studies

**DOI:** 10.3389/fnut.2021.787908

**Published:** 2022-01-21

**Authors:** Nansi López-Valverde, Antonio López-Valverde, Bruno Macedo de Sousa, Cinthia Rodríguez, Ana Suárez, Juan Manuel Aragoneses

**Affiliations:** ^1^Department of Surgery, University of Salamanca, Salamanca, Spain; ^2^Instituto de Investigación Biomédica de Salamanca (IBSAL), Salamanca, Spain; ^3^Institute for Occlusion and Orofacial Pain Faculty of Medicine, University of Coimbra, Coimbra, Portugal; ^4^Department of Dentistry, Universidad Federico Henríquez y Carvajal, Santo Domingo, Dominican Republic; ^5^Department of Preclinical Dentistry, School of Biomedical Sciences, Universidad Europea de Madrid, Madrid, Spain; ^6^Faculty of Dentistry, Universidad Alfonso X El Sabio, Madrid, Spain

**Keywords:** probiotics, halitosis, oral malodor, bad breath, oral health, randomized clinical trial

## Abstract

Halitosis or oral malodor is a condition caused by the putrefaction of sulfur-containing amino acids. It affects 30–50% of the population and causes social rejection, reducing quality of life, and self-esteem. Probiotics, especially Lactobacillus species, have been proposed for the treatment of genuine halitosis, due to their ability to reduce bacterial colonization. Our objective was to evaluate their use for the treatment of oral halitosis. Applying the PRISMA statement guidelines, we searched PubMed, EMBASE, and Web of Science databases for scientific articles from the last 15 years, up to July 2021. The keywords used were “Probiotics”; “Halitosis”; “Mouth diseases”; “Oral health”; “Humans”; “Randomized Clinical Trials” according to the question, “Are probiotics effective for the reduction or elimination of oral halitosis?” Fourteen studies were identified, although only four met the inclusion criteria. We evaluated 283 participants treated with two different probiotics, with a follow-up of at least 2 weeks. Risk of bias was assessed using the Cochrane Collaboration tool. A fixed-effects meta-analysis was performed. No statistical significance was found (*p* = 0.53). Despite the limitations of this meta-analysis, we believe that some probiotics have a beneficial effect on halitosis, although more clinical trials are needed to establish real evidence on this aspect.

**Systematic Review Registration:** https://doi.org/10.37766/inplasy2021.9.0009, identifier: INPLASY20211900.

## Introduction

Halitosis, or bad breath, is a term referring to an unpleasant or annoying odor that emanates from the oral cavity of an individual and affects between 30 and 50% of the population (1). Its main cause is the putrefaction of sulfur-containing amino acids, with hydrogen sulfide, methylmercaptan, and dimethylsulfide being the main culprits (2). Occasional halitosis may be associated with the consumption of certain condiments or food seasonings, such as onion or garlic, or with certain pathologies such as uremia or diabetes (3, 4).

The International Society for Breath Odor Research (ISBOR) classifies halitosis according to its origin into three categories: genuine halitosis, pseudohalitosis, and halitophobia. Genuine halitosis can occur under certain pathological conditions, or in a physiological form, in which the malodor originates in the oral cavity and is not associated with a specific pathological condition or disease. This type of halitosis exhibits a bad breath odor that exceeds the socially acceptable level, produced by putrefaction processes in the oral cavity, without the existence of a specific disease or pathological condition that causes it, being its main origin, the plaque (biofilm) accumulated in the interdental spaces, the dorsoposterior region of the tongue, under the prosthesis or in the orthodontic appliances (5, 6).

Halitosis has a great psychological impact due to the social isolation it produces, being one of the conditions for which people seek dental care, after caries and periodontal disease (7, 8). However, it should be noted that people with chronic halitosis minimize contact with their social environment, resulting in a low quality of life and decreased self-esteem, precisely because of the social difficulties in relating to others, even avoiding intimacy, leading to the search for medical help, precisely because of the social consequences that their condition entails (7, 9).

Probiotics are non-pathogenic live microorganisms, which are administered to improve microbial balance, especially in the gastrointestinal tract (10). They consist of yeasts or lactic acid bacteria, such as *Lactobacillus* species, and are regulated as dietary supplements and foods. They exert their beneficial effects through several mechanisms, such as reduction of intestinal pH, reduction of bacterial colonization and invasion of pathogenic organisms, along with modification of the host immune response (11). The most commonly used are lactic acid bacteria, specifically *Lactobacillus* and *Bifidobacterium* species (12). In general, they are used for the prevention and treatment of various medical conditions and to support general well-being.

**Figure 1:**
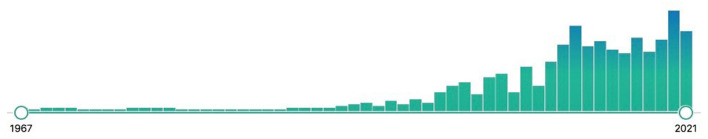
Publications in the US National Library of Medicine database with the following keywords: “Halitosis” and “Oral Health.” Source: US National Library of Medicine (17).

In recent years, certain studies have proposed them to control plaque formation and prevent the breakdown of microbial homeostasis, acting on the maintenance and improvement of oral health (13–16). Figure 1 presents a graph demonstrating the increase in publications over the last 20 years (prepared using data from the US National Library of Medicine) (17).

The objective of this systematic review with meta-analysis was to evaluate the efficacy of probiotics in genuine halitosis of oral origin.

## Materials and Methods

### Study Design

A bibliographic search was conducted following the PRISMA [Preferred Reporting Items for Systemic Reviews and Meta-Analyses http://www.prisma-statement.org (accessed on 5 June, 2021)] guidelines for systematic reviews and meta-analyses INSPLAY registration number: INPLASY202190009. The review also fulfilled the PRISMA 2015 Checklist (18) (Supplementary Table 1).

### Question of Interest

The PICO (population, intervention, comparison, and outcome) question was “Are probiotics effective for the reduction or elimination of oral halitosis?” with the following components: population-subjects suffering from halitosis; intervention-probiotic administration; control-subjects not receiving probiotics; outcome-reduction of halitosis.

### Data Sources and Search Strategy

The PubMed, EMBASE and Web of Science (WOS) electronic databases were searched for findings published, in the last 15 years until July 2021. The MeSH (Medical Subject Headings) terms used in the MEDLINE (PuBMed) databases were: “Probiotics” [MeSH terms], “Halitosis” [MeSH terms], “Mouth diseases” [MeSH terms], “Oral health” [MeSH terms], “Humans” [MeSH terms], “Randomized Clinical Trials” [MeSH terms]; the Boolean operator AND was used to refine the search. The search terms used in Embase were: “Probiotics,” “Halitosis,” “Randomized Clinical Trials.” In WOS, the search terms were: “Probiotics,” “Halitosis,” “Oral health,” “Randomized Clinical Trials”; the Boolean operators AND-OR were used to refine the search.

### Inclusion and Exclusion Criteria

The inclusion criteria for the study selection were: Articles published in English; Randomized clinical studies referring to the benefits of probiotics on oral halitosis; Follow-up for at least 2 weeks.

The exclusion criteria for the study selection were: Animal studies; *In vitro* studies; Narrative and systematic reviews; Studies on the benefits of probiotics in other types of oral pathologies; Clinical cases duplicate and informative studies, in languages other than English or with a follow-up of <2 weeks.

### Data Extraction and Analysis

Studies that did not address the research question were eliminated, and the titles and abstracts of the selected articles were collected and entered into an Excel spreadsheet. Two reviewers (NL-V and AL-V) independently selected the titles and abstracts. Disagreements about the inclusion of studies were resolved by discussion between the two reviewers. Subsequently, the full texts of the selected studies were obtained for review and inclusion. The bibliographic references of each study were also reviewed as possible sources for identifying additional studies.

### Quality and Risk of Bias Assessments

The methodological quality and risk of bias of each eligible trial were independently assessed using the Cochrane Collaboration tool for assessing risk of bias in randomized trials by two investigators (19). Any discrepancies were resolved through discussion with a third investigator.

### Statistical Analysis

The meta-analysis was performed using RevMan software [Review Manager (RevMan) (Computer program). Version 5.4.1, The Cochrane Collaboration, 2020]. Given the homogeneity of the studies, a fixed effects model was used; the mean difference (MD) and standard deviation (SD) were used to evaluate the continuous variable (probiotic) with a 95% confidence interval (CI).

## Results

### Characteristics of the Studies

**Figure 2:**
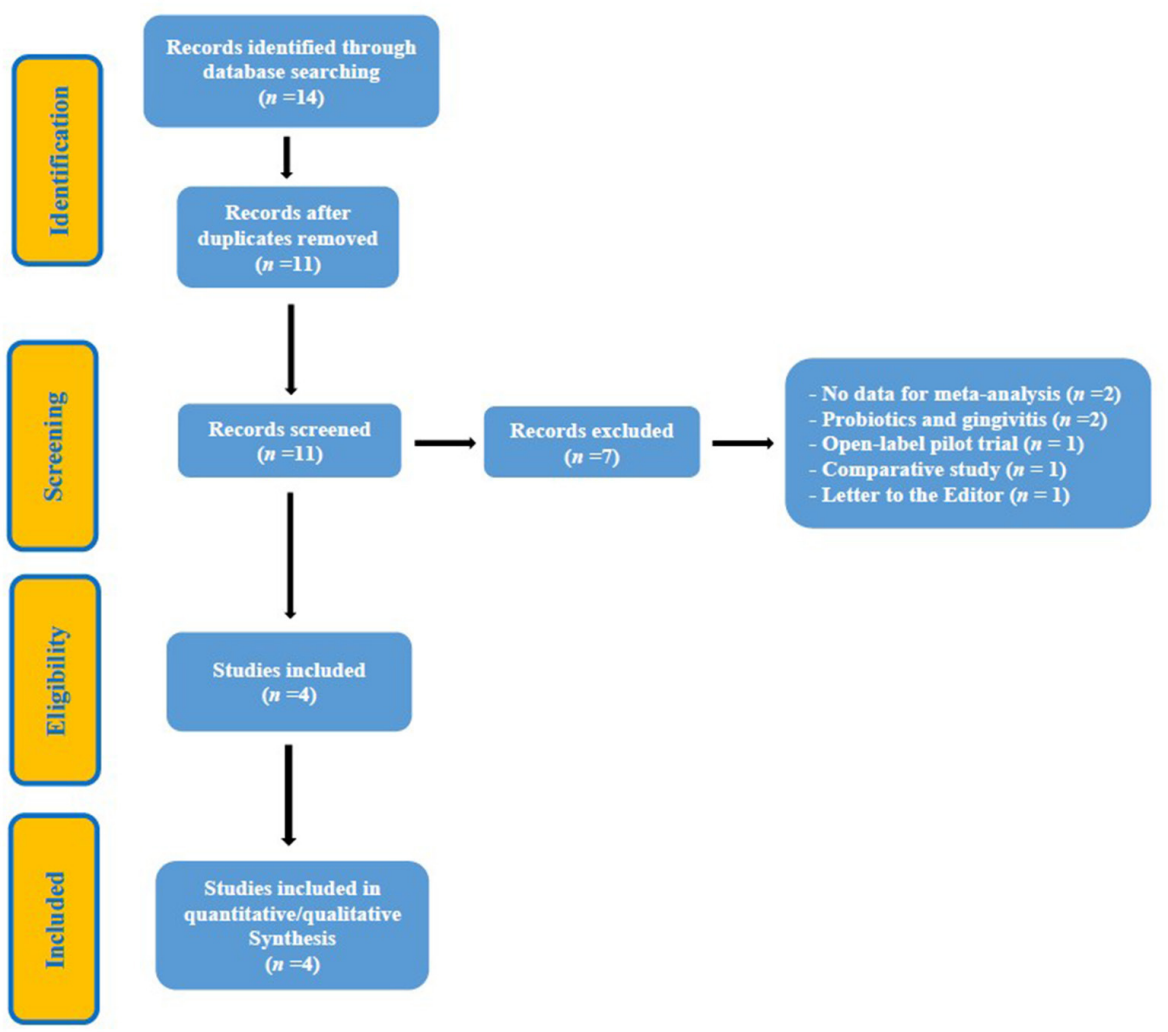
Flowchart.

Until July 2021, a total of 14 studies were identified and subsequently evaluated by the reviewers. After a first screening, three duplicate studies were eliminated. A second evaluation led to the elimination of seven studies, which were considered inappropriate because they did not clearly meet the inclusion criteria. Finally, four studies were selected that met the inclusion criteria in full (20–23) (Figure 2, Flowchart). A total of 283 participants were evaluated and the longest follow-up was 12 weeks (20, 21). Three of the studies (20–22) used *Streptococcus salivarius* (SS), strains K12 and M18, as a probiotic and one (23) used *Weissella cibaria* (WC). Three studies evaluated the Organoleptic Test (OLT) and Volatile Sulfur Compounds Levels (VSCLs) (20, 22, 23) and one (21), exclusively, the latter parameter. The study by Lee et al. (23) was the only one to measure the Bad Breath (BB) improvement scores. Tables 1, 2 provide a summary of the details of the RCTs.

**Table 1:** General characteristics of studies.

**References**	**Study type**	**Participants (*n*)**	**Follow-up (weeks)**	**Probiotic type**	**Study conclusions**
Jamali et al. (20)	Randomized Controlled Clinical Trial	*n* = 99	12 weeks	SS K12	Probiotic therapy following oral disinfection with chlorhexidine may reduce the severity of halitosis over longer periods.
Benic et al. (21)	Randomized, triple-blind, placebo-controlled trial	*n* = 64	12 weeks	SS M18	Oral probiotic SS M18 reduced the level of halitosis in patients wearing orthodontic.
He et al. (22)	Randomized, placebo-controlled trial	*n* = 28	4 weeks	SS K12	The use of SS K12 did not have significant effect on halitosis with tongue coating cause when the tongue coating was not physically or chemically pre-treated, which implies removing tongue coating is required before SS K12 use.
Lee et al. (23)	Randomized, double-blind, placebo-controlled study	*n* = 92	8 weeks	WC	WC tablets could prove to be a safe and useful oral care product to control bad breath.

*SS, Streptococcus salivarius, WC, Weissella cibaria*.

**Table 2:** Patients and parameters measured in the included studies.

**References**	**Type of patient**	**Parameters measured**	**Age range (years)**	**Measurement system**	**Outcomes**
Jamali et al. (20)	Children	OLT	6–9	Judge examiner	A significant and stable number of participants showed moderate levels of improvement in organoleptic tests in the experimental group (probiotic + chlorhexidine) vs. the control group.
Benic et al. (21)	Children/ Adults	VSCLs	10–30	Halimeter®	VSCLs decreased significantly in the probiotic-treated group at 3-month follow-up, while in the placebo-treated group, VSCLs returned to baseline levels.
He et al. (22)	Adults	OLT and VSCLs	23–44	Halimeter®	Both groups (test and control) showed a significantly lower OLT score compared to baseline values. VSCLs in the test group the decrease was statistically significant.
Lee et al. (23)	Adults	OLT, VSCLs and BB	20–39	Judge examiner	A significant decrease in OLT and VSCLs scores was observed in the test group, while BB scores decreased significantly.

*OLT, Organoleptic test; VSCLs, Volatile Sulfur Compound Levels; BB, Bad Breath*.

### Quality and Risk of Bias

**Figure 3:**
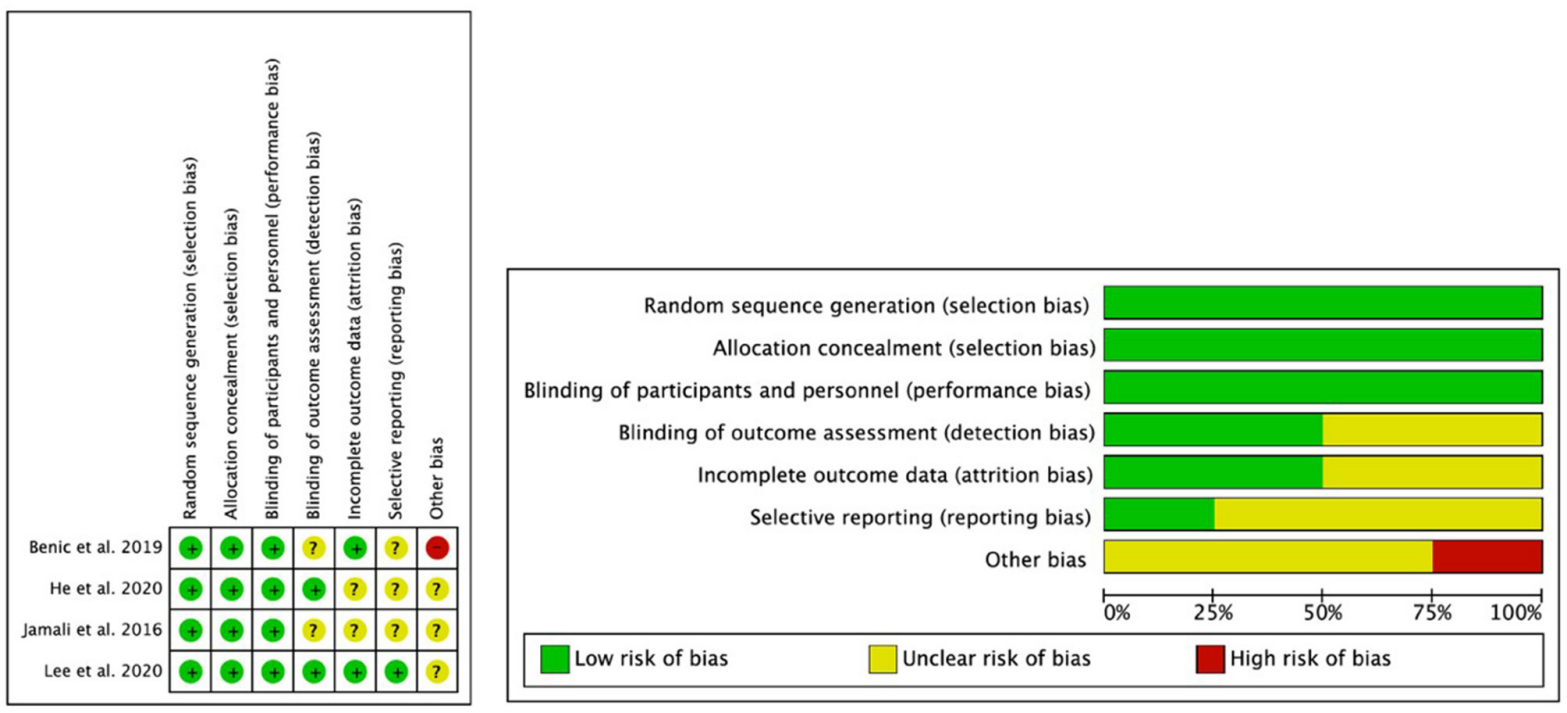
The Cochrane Collaboration's tool for assessing the risk of bias of included randomized controlled trials.

The methodological quality and risk of bias of each eligible study were evaluated using the Cochrane Collaboration tool for assessing the risk of bias, and the results are presented in Figure 3. All studies were randomized. All studies met the items Random sequence generation, Allocation concealment and Blinding of participants and staff; the item Selective reporting was the item with the least clear risk of bias. The study by Jamali et al. (20), showed the highest number of unclear items.

### Meta-Analysis Results

All included studies measured the effect of different probiotics on genuine halitosis. A fixed-effects meta-analysis was performed, based on the results of four studies. The overall results showed homogeneity (*I*^2^ = 0%, CI 95%). The studies by Jamali et al. (20) and Lee et al. (23) had the highest weight in the meta-analysis; the study by He et al. (22), despite having a much lower weight than the other studies (9.8%) did not have a high risk of bias, so we did not consider it necessary to perform a sensitivity analysis.

**Figure 4:**
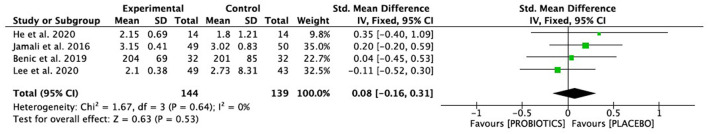
Forest Plot of the effectiveness on halitosis of probiotic vs. placebo, in a fixed effects model. SD, Standard Deviation, CI, Confidence Interval.

No statistical significance was found between the intervention group and the control group (*p* = 0.53) (Figure 4).

### Publication Bias and Heterogeneity

**Figure 5:**
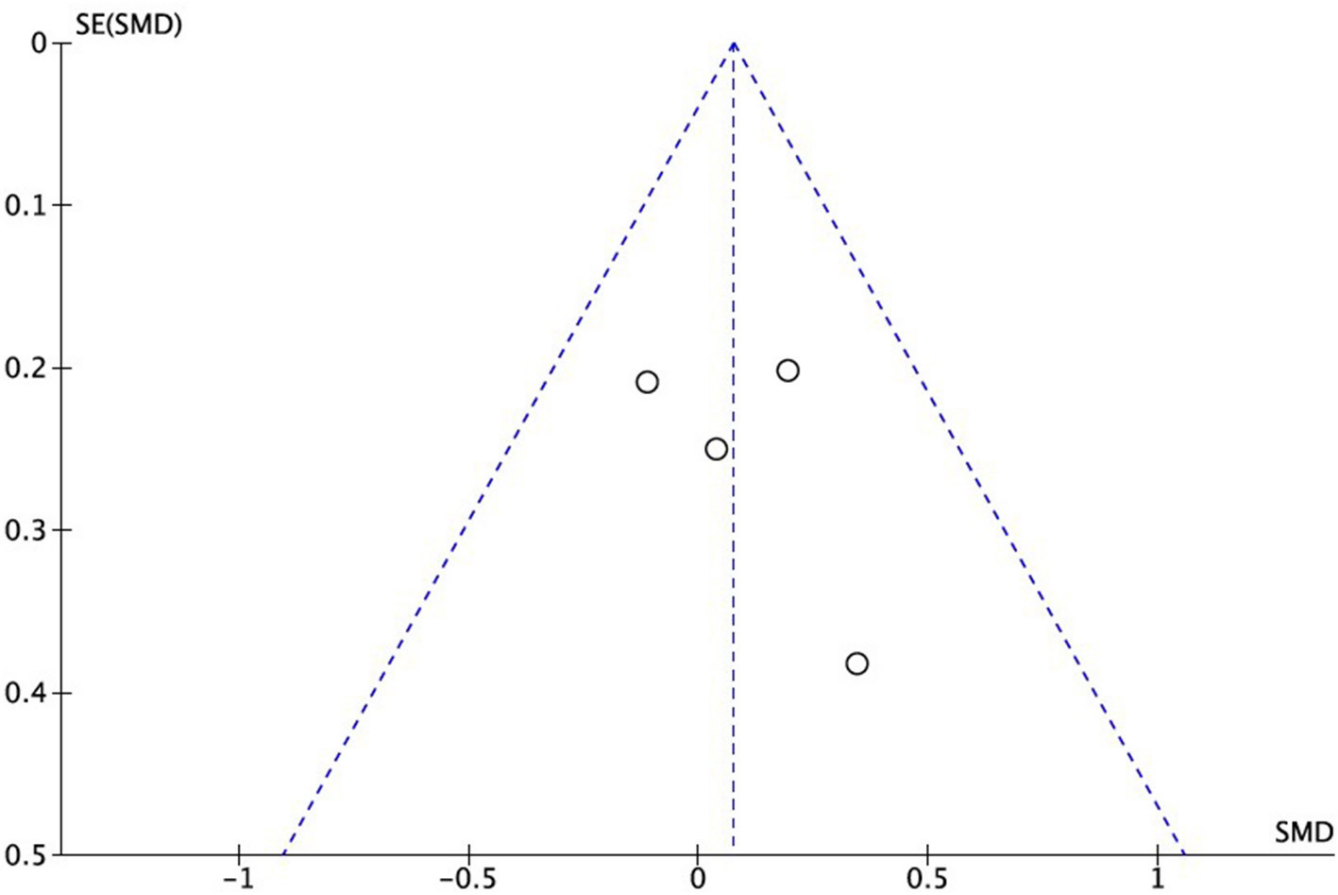
Funnel Plot of publication bias.

The RCTs included in the meta-analysis showed no graphic signs of publication bias, as can be seen in the Funnel Plot in Figure 5.

## Discussion

Oral health is a vital component of public health. The World Health Organization defines it as a state free of chronic pain in the mouth and face, throat cancer, oral sores, birth defects such as cleft lip and palate, periodontal diseases, dental caries, tooth loss, and other diseases and disorders affecting the oral cavity (24). Halitosis of any kind is a public health problem, especially because of the social rejection it entails (7).

The term probiotic was first used in the 1960s (25). Since then, bacteriotherapy has led to alternative ways of fighting certain infectious diseases, with fewer side effects than conventional drugs. During the last few years, the use of probiotics has gained interest among the dental community, developing studies focused on the reduction of different pathologies of the oral cavity, such as the incidence of dental caries, periodontitis, halitosis, or infections such as oral candidiasis. The modulation of the microbiota through the use of probiotics or new generation beneficial microbes, constitutes a future perspective in the development of pharmaceutical tools to maintain health (26, 27), however, randomized clinical studies are scarce, most of them being reduced to reviews of the scientific literature.

Halitosis is mainly attributed to biofilm accumulation on the dorsum of the tongue, interdental spaces, under orthodontic and orthopedic prostheses and appliances, and chronic inflammatory diseases of the periodontium. Self-care products, such as chewing gums, mouth rinses and sprays, and toothpastes, can temporarily mask oral odor and improve the individual's satisfaction and self-esteem; however, the proper approach remains professional treatment and appropriate strategies for the reduction and/or elimination of the microorganism-producing causes (28, 29).

Although De Boever and Loesche (30) had suggested that the proteolytic microbiota colonizing the tongue, contributes greatly to halitosis by its action of degrading food proteins, and therefore a probiotic strain colonizing the tongue surface and not producing odorous metabolic by-products would be well-suited to control halitosis, it was Burton et al. who addressed the treatment of halitosis by probiotics (31, 32) in a study of 23 subjects subjected to chlorhexidine mouth rinses and the use of SS K12 tablets vs. placebo, demonstrating a significant reduction in halitosis levels. In the same aspect, Jamali et al. (20), highlighted the importance of pre-rinse with chlorhexidine before probiotic use.

Different probiotics have been used in the treatment of halitosis; two of the included studies included in our meta-analysis used SS K12 (20, 22). In fact, SS is known to be a pioneer colonizer of oral surfaces and predominant in the saliva of non-halitosis subjects (33), thus, it is considered to have excellent potential for use as a probiotic, targeting halitosis-producing bacteria. Masdea et al. in an *in vitro* study (34), demonstrated the antimicrobial activity of SS K12 against several bacteria responsible for halitosis, including *Solobacterium moorei*, which has been described as one of the main culprits of oral malodor.

The study by Lee et al. (23) used WC as a probiotic. This is a Gram-positive bacterium, widely represented in saliva or fermented foods, which exhibits preventive effects on the formation of biofilms and the production of volatile sulfur compounds, which would suggest its use as a probiotic in oral halitosis care (35–37).

Yoo et al. (38) and Meurman and Stamatova (39) studied *in vitro* the antimicrobial activity of SS strain M18 against the bacteria causing oral malodor; Benic et al. (21) in a randomized study on a sample of 32 patients with orthodontic appliances demonstrated the effectiveness of the oral probiotic SS M18, which reduced the level of halitosis, although it had minimal effects on the plaque index and dental biofilm microflora.

Similarly, we have to consider that bad breath can vary between children and adults, throughout the day, before and after menstruation in women, and at the time of starting to speak (40–42).

On the other hand, measurement systems can lead to confusing results. The studies included in this meta-analysis used different measurement systems; Jamali et al. (20) and Lee et al. (23) used the OLT using a judge examiner and the Greenman test (43), however, Benic et al. (21) and He et al. (22) used measuring devices, such as the Halimeter®, Interscan, USA (44).

The OLT is the simplest method of measuring oral malodor by “human judges” and reflects an everyday situation and despite being considered the gold standard for measuring bad breath, it is quite embarrassing for both (examiner and examinee). Both the reliability and reproducibility of this method are problematic. Concerning the agreement between “judges,” this can be improved by standardizing the sense of smell, using different odor solution kits (e.g., T&T Olfactometer®, Daiichi Yakuhin Sangyo, Tokyo, Japan). At the same time, for better inter-judge agreement, patients should refrain from oral hygiene practices, smoking, ingesting antibiotics and meals with garlic, onion, and spices before the measurement; moreover, inter-judge agreement increases if they themselves also avoid drinking coffee and tea, smoking, and using cosmetics with odor before the organoleptic tests (45).

Currently, given the development of new technologies that identify and measure the compounds present in the breath and the discomfort experienced by both (patient and health professional), OLT are increasingly out of use, with the use of monitors for measuring VSCLs, such as Halimeter®, which measures total volatile sulfur compounds, although it does not identify them, and therefore is not in itself a sufficient diagnostic method to evaluate the existence of halitosis, although it is the most reliable method, since it separates the gases by their molecular weight and allows their identification and measurement in samples of exhaled air, saliva, feces, etc. (46).

Gas chromatography can be combined with mass spectrometry, thus increasing the range of this method. It is considered a highly objective, reproducible, and reliable method. However, few clinics have this system implemented, due to its high cost, the need for highly qualified and trained professionals to handle it, and the long duration of its procedures (47). ISBOR (International Society for Breath Odor Research) is making an effort for the creation and maintenance of a database, which already includes more than 3,000 registered volatile compounds (48).

The present systematic review had several limitations. Firstly, the small number of studies included in the meta-analysis; secondly, some of the studies complemented the use of probiotics with the use of an antiseptic (chlorhexidine) (20), with the consequent bias in the results; third, another of the included studies was performed in patients with orthodontic appliances (21), with the consequent increase in bacterial plaque retention caused by this type of appliance; fourth, there were different follow-up periods in the studies (4, 8, and 12 weeks) and different parameters indicative of oral malodor were measured; fifth, different probiotics (SS K12, M18 and WC) were used. Therefore, the results obtained should be taken with caution.

## Conclusions

The lack of statistical significance (*p* = 0.53) cannot point in a particular direction, as the term “statistically significant” does not mean having relevant effects, as a statistically significant association may not be clinically or epidemiologically relevant. Therefore, we believe that some probiotics appear to have a beneficial effect on halitosis of oral origin, however, multicenter clinical trials with sufficient statistical power and the use of high-throughput analytical tools are needed to establish real evidence of the role of probiotics in oral health and to justify their use in halitosis. At present, existing trials remain scarce.

## Data Availability Statement

The original contributions presented in the study are included in the article/Supplementary Material, further inquiries can be directed to the corresponding author/s.

## Author Contributions

NL-V and AL-V: conceptualization, writing, review, and editing. JA and BM: methodology. AL-V, CR, AS, and JA: validation. NL-V: formal analysis. NL-V and BM: data curation. AL-V: supervision. All authors have read and agreed to the published version of the manuscript.

## Conflict of Interest

The authors declare that the research was conducted in the absence of any commercial or financial relationships that could be construed as a potential conflict of interest.

## Publisher's Note

All claims expressed in this article are solely those of the authors and do not necessarily represent those of their affiliated organizations, or those of the publisher, the editors and the reviewers. Any product that may be evaluated in this article, or claim that may be made by its manufacturer, is not guaranteed or endorsed by the publisher.
